# National guideline development and adaptation, Egypt

**DOI:** 10.2471/BLT.24.293024

**Published:** 2026-03-30

**Authors:** Mehrnaz Kheirandish, Malika A Tamim, Arash Rashidian, Riham El-Asady, Naeema Al Qasseer, Gasser Gad El-Kareem, Mohamed Mostafa Lotayef, Mohammed Mourad Youssif, Elie A Akl, Nathan Ford, Paul Garner

**Affiliations:** a Division of Health Systems and Life Course, World Health Organization Regional Office for the Eastern Mediterranean, Monazamet El Seha El Alamia Street, Cairo, 11371, Egypt.; b World Health Organization Country Office, Cairo, Egypt.; c Egyptian Health Council, Cairo, Egypt.; d National Guideline Development and Adaptation Programme, Cairo, Egypt.; e Department of Internal Medicine, American University of Beirut, Beirut, Lebanon.; f Department for HIV, Tuberculosis, Hepatitis and Sexually Transmitted Infections, World Health Organization, Geneva, Switzerland.; g Department of Clinical Sciences, Liverpool School of Tropical Medicine, Liverpool, England.

## Abstract

**Problem:**

In 2018, the Egyptian government enacted a universal health coverage law mandating provision of high-quality, effective and equitable health care. Achieving this mandate would require expanding availability and use of evidence-based national clinical practice and public health guidelines.

**Approach:**

In 2022, Egypt's Ministry of Health and Population requested support from the World Health Organization (WHO) to establish a national guideline development and adaptation programme. To inform the process, WHO conducted a situation analysis. The findings led to a collaboration between WHO and national stakeholders including the newly established Egyptian Health Council. In partnership they delivered capacity-building, developed a roadmap and implemented the programme.

**Local setting:**

Egypt is a lower-middle income country of 117 million people. Health services are provided by agencies in public (governmental and semi-governmental) and private sectors (providers and professional syndicates). No national guideline programme existed before 2022.

**Relevant changes:**

In total, 225 individuals attended the capacity-building workshops. After the council ratified the roadmap, the ministry, WHO and the council formally launched the national guideline programme in July 2024. Implementation of the programme led to clearer delineation of responsibilities, eliminated role overlaps and harmonized processes across different professional societies, teaching hospitals, nurses and allied health professionals.

**Lessons learnt:**

Consistent, informed political support and stakeholder commitment contributed to successful implementation. WHO’s approach to contextualizing global guidelines added value. Challenges included reliance on international experts, constrained resources and insufficient institutional capacity. Moving forward, sustained institutional development, coaching, monitoring and evaluation, funding and stakeholder engagement are needed.

## Résumé

**Problème:**

En 2018, le gouvernement égyptien a promulgué une loi sur la couverture sanitaire universelle, imposant la fourniture de soins de santé efficaces, équitables et de haute qualité. Pour mener à bien cette mission, il y a lieu d’élargir la disponibilité et l’utilisation de lignes directrices nationales fondées sur des données probantes en matière de pratique clinique et de santé publique.

**Approche:**

En 2022, le ministère égyptien de la Santé et de la Population a sollicité l’aide de l’Organisation mondiale de la Santé (OMS) pour mettre en place un programme national d’élaboration et d’adaptation de ces lignes directrices. Afin d’éclairer ce processus, l’OMS a procédé à une analyse de la situation. Ses conclusions ont donné lieu à une collaboration entre l’OMS et les parties prenantes à l’échelle nationale, notamment le Conseil égyptien de la santé nouvellement créé. Elles ont mis en place un partenariat pour assurer le renforcement des capacités, élaborer une feuille de route et mettre en œuvre le programme.

**Environnement local:**

L’Égypte est un pays à revenu intermédiaire inférieur comptant 117 millions d’habitants. Les services de santé y sont fournis par des organismes des secteurs public (gouvernementaux et semi-gouvernementaux) et privé (prestataires et syndicats professionnels). Il n’existait aucun programme national de lignes directrices avant 2022.

**Changements pertinents:**

En tout, 225 personnes ont participé aux ateliers de renforcement des capacités. Après la ratification de la feuille de route par le Conseil, ce dernier, le ministère et l’OMS ont officiellement lancé le programme national de lignes directrices en juillet 2024. La mise en œuvre du programme a permis de mieux délimiter les responsabilités, d’éliminer les chevauchements des postes et d’harmoniser les processus entre les différentes associations professionnelles, les hôpitaux universitaires, le personnel infirmier et les professionnels de santé dans le domaine paramédical.

**Leçons tirées:**

Un soutien politique cohérent et éclairé ainsi que l’engagement des parties prenantes ont contribué à la réussite de la mise en œuvre du programme. L’approche de l’OMS visant à contextualiser les lignes directrices mondiales y a apporté une valeur ajoutée. Parmi les défis figuraient la dépendance vis-à-vis d’experts internationaux, des ressources limitées et une capacité institutionnelle insuffisante. Par la suite, un développement institutionnel soutenu, un accompagnement, un suivi et une évaluation, un financement et la participation des parties prenantes seront nécessaires.

## Resumen

**Situación:**

En 2018, el Gobierno de Egipto promulgó una ley de cobertura sanitaria universal que exige la provisión de una asistencia sanitaria de alta calidad, eficaz y equitativa. El cumplimiento de este mandato requiere ampliar la disponibilidad y el uso de guías nacionales de práctica clínica y de salud pública basadas en la evidencia.

**Enfoque:**

En 2022, el Ministerio de Salud y Población de Egipto solicitó apoyo a la Organización Mundial de la Salud (OMS) para establecer un programa nacional de desarrollo y adaptación de guías. Para fundamentar el proceso, la OMS realizó un análisis de situación. Los resultados dieron lugar a una colaboración entre la OMS y las partes interesadas nacionales, incluido el Consejo de Salud Egipcio recientemente establecido. En colaboración, se llevaron a cabo actividades de fortalecimiento de capacidades, se elaboró una hoja de ruta y se implementó el programa.

**Marco regional:**

Egipto es un país de ingreso mediano bajo con una población de 117 millones de personas. Los servicios sanitarios son prestados por entidades de los sectores público (gubernamental y semigubernamental) y privado (proveedores y colegios profesionales). Antes de 2022 no existía un programa nacional de guías.

**Cambios importantes:**

En total, 225 personas participaron en los talleres de fortalecimiento de capacidades. Tras la ratificación de la hoja de ruta por el consejo, el ministerio, la OMS y el propio consejo lanzaron formalmente el programa nacional de guías en julio de 2024. La implementación del programa permitió una delimitación más clara de responsabilidades, eliminó la superposición de funciones y armonizó los procesos entre distintas sociedades profesionales, hospitales docentes, personal de enfermería y otros profesionales sanitarios.

**Lecciones aprendidas:**

El apoyo político sostenido e informado, así como el compromiso de las partes interesadas, contribuyeron a una implementación satisfactoria. El enfoque de la OMS para contextualizar las guías globales aportó valor añadido. Entre los desafíos se incluyeron la dependencia de expertos internacionales, la limitación de recursos y la insuficiente capacidad institucional. De cara al futuro, se requieren el desarrollo institucional sostenido, el acompañamiento técnico, el seguimiento y la evaluación, la financiación y la implicación de las partes interesadas.

## ملخص

**المشكلة:**

في عام 2018، قامت الحكومة المصرية بسن قانون للتغطية الصحية الشاملة يشترط توفير رعاية صحية عالية الجودة وفعّالة وعادلة. ويتطلب تحقيق هذا الهدف توسيع نطاق توافر واستخدام الممارسات السريرية الوطنية القائمة على الأدلة والمبادئ التوجيهية للصحة العامة.

**الأسلوب:**

في عام 2022، طلبت وزارة الصحة والسكان المصرية الدعم من منظمة الصحة العالمية (WHO) لوضع برنامج وطني لتطوير وتكييف المبادئ التوجيهية. ولإثراء هذه العملية، أجرت منظمة الصحة العالمية تحليلًا للوضع الراهن. وأدت النتائج إلى تعاون بين منظمة الصحة العالمية والجهات المعنية الوطنية، بما في ذلك المجلس الصحي المصري حديث التأسيس. ومن خلال الشراكة، قدمت هذه الجهات حلولاً لبناء القدرات، ووضعت خارطة طريق، وقامت بتنفيذ البرنامج.

**المواقع المحلية:**

مصر دولة ذات دخل متوسط منخفض، ويبلغ عدد سكانها 117 مليون نسمة. يتم تقديم الخدمات الصحية من جانب جهات في القطاعين العام (الحكومي وشبه الحكومي)، والخاص (مقدمي الخدمات والنقابات المهنية). لم يكن هناك برنامج وطني للمبادئ التوجيهية قبل عام 2022.

**التغيّرات ذات الصلة:**

في المجمل، حضر 225 شخصًا ورش عمل لبناء القدرات. بعد مصادقة المجلس على خارطة الطريق، أطلقت الوزارة ومنظمة الصحة العالمية والمجلس رسميًا برنامج المبادئ التوجيهية الوطنية في يوليو/تموز 2024. وقد أدى تنفيذ البرنامج إلى تحديد أوضح للمسؤوليات، والقضاء على التداخل بين الأدوار، وتنسيق العمليات بين مختلف الجمعيات المهنية، والمستشفيات التعليمية، والممرضين والمتخصصين في المهن الصحية المساندة.

**الدروس المستفادة:**

ساهم الدعم السياسي المتواصل والواعي والتزام أصحاب المصلحة في نجاح التنفيذ. كما أضاف أسلوب منظمة الصحة العالمية قيمة في صياغة المبادئ التوجيهية. وشملت التحديات الاعتماد على الخبراء الدوليين، ومحدودية الموارد، وعدم كفاية القدرات المؤسسية. وللمضي قدمًا، فإن هناك حاجة إلى التطوير المؤسسي المستدام، والتدريب، والرصد والتقييم، والتمويل، وإشراك أصحاب المصلحة.

## 摘要

**问题:**

埃及政府于 2018 年颁布了全民健康覆盖法，旨在强制要求提供优质、有效且公平的医疗保健服务。如要实现该目标，将需扩大循证国家临床实践和公共卫生指南的可获得性和使用范围。

**方法:**

埃及卫生和人口部于 2022 年请求世卫组织 (WHO) 提供相应支持以构建国家指南制定与改编计划。为了指导流程推进，世卫组织分析了具体情况。根据分析结果，世卫组织和国家利益攸关方（包括新成立的埃及卫生理事会）决定联手合作。在通力合作下，他们开展了能力建设、制定了路线图并实施了该计划。

**当地状况:**

埃及是一个拥有 1.17 亿人口的中等偏下收入国家。卫生服务由公共部门（政府部门和半政府部门）和私营部门（提供商和专业行业协会）的相关机构共同提供。在 2022 年之前并未实施任何国家指南计划。

**相关变化:**

总计有 225 人参加了能力建设研讨会。卫生理事会批准路线图后，卫生和人口部、世卫组织及卫生理事会于 2024 年 7 月正式推出了国家指南计划。随着计划实施，责任划分变得更加明确，职责重叠现象得以消除，且不同专业协会、教学医院、护士和医务辅助人员之间的工作流程也实现了协调同步。

**经验教训:**

持续而明智的政治支持以及利益攸关方的全力投入，保障了该计划的成功实施。世卫组织因地制宜地改编全球指南的方法发挥了增值效应。但是计划实施过程中也存在各种挑战，包括依赖于国际专家、资源有限及机构能力不足。未来还需持续推进机构发展、提供专业指导、进行监督与评估、提供资金支持和确保利益攸关方参与其中。

## Резюме

**Проблема:**

В 2018 году Правительство Египта приняло закон о всеобщем охвате услугами здравоохранения, которым предусматривается предоставление высококачественной, эффективной и справедливой медицинской помощи. Для достижения этой цели требовалось расширение доступности и использования национальной клинической практики и руководств в области общественного здравоохранения, основанных на доказательных принципах.

**Подход:**

В 2022 году Министерство здравоохранения и народонаселения Египта обратилось к Всемирной организации здравоохранения (ВОЗ) с просьбой о поддержке в создании программы разработки и адаптации национальных руководств. Для получения необходимой для этого процесса информации ВОЗ провела ситуационный анализ, по результатам которого Всемирная организация здравоохранения начала сотрудничать с национальными заинтересованными сторонами, в том числе с недавно созданным Египетским советом по вопросам здравоохранения. В рамках партнерства были проведены мероприятия по наращиванию потенциала, разработана дорожная карта и реализована программа.

**Местные условия:**

Египет – это страна с населением 117 млн человек со средненизким уровнем дохода. Услуги здравоохранения предоставляются учреждениями государственного (правительственного и полуправительственного) и частного секторов (поставщиками услуг и профессиональными ассоциациями). До 2022 года программа национальных руководств отсутствовала.

**Осуществленные перемены:**

Семинары по наращиванию потенциала посетили в общей сложности 225 человек. После того как совет ратифицировал дорожную карту, Министерство, ВОЗ и совет официально запустили программу национальных руководств в июле 2024 года. Реализация программы привела к более четкому разграничению ответственности, устранению дублирования функций и согласованию процессов между различными профессиональными сообществами, учебными больницами, сестринским персоналом и специалистами смежных медицинских областей.

**Выводы:**

Последовательная и информированная политическая поддержка, а также приверженность заинтересованных сторон способствовали успешной реализации. Дополнительную ценность придал подход ВОЗ к контекстной адаптации глобальных руководств. Среди проблем можно отметить зависимость от международных экспертов, ограниченность ресурсов и недостаточный институциональный потенциал. Для дальнейшего прогресса необходимы: устойчивое институциональное развитие, наставничество, мониторинг и оценивание, а также финансирование и вовлечение заинтересованных сторон.

## Introduction

Clinical practice and public health guidelines are documents assisting health workers and patients in deciding on appropriate health care. These documents also inform decision-makers about policies and strategies that can help prevent disease, promote population well-being and improve health outcomes.^1^ Developing appropriate guidelines, which often include systematically developed, evidence-based recommendations, is costly, time consuming and requires institutions with strong technical capacities.^2^^,^^3^ Since international guidelines often require modifications to fit local contexts, many countries establish guideline adaptation programmes to ensure a thorough and rigorous process.^2^

Most countries in the World Health Organization (WHO) Eastern Mediterranean Region lack established national guideline programmes.^4^^,^^5^ In 2019, the 66th Session of WHO’s Regional Committee for the Eastern Mediterranean approved an approach for enhancing national institutional capacity for evidence-informed policy-making, including the establishment of national guideline programmes.^6^

In January 2018, the Egyptian government enacted a universal health insurance law to provide universal health coverage to its population within 10 years. The law mandated high-quality, effective and equitable health care and aimed to reduce variations in the provision and access to care. However, the many national clinical practice and public health guidelines needed to achieve these objectives were either lacking or of low quality. Here we describe the efforts made to improve guideline quality, reduce inconsistencies and ultimately improve health outcomes.

## Local setting

Egypt is a lower-middle income country with a population of 117 million. Health services are provided by agencies in public (governmental and semi-governmental) and private sectors (providers and professional syndicates). There have been considerable efforts to reform the health system since 2015. 

## Approach

In 2022, the Minister of Health and Population sought WHO’s assistance to establish a national guideline development and adaptation programme. The request initiated extensive collaboration between WHO and two main stakeholders with relevant policy-making roles: the Egyptian Health Council and the Ministry of Health and Population. The Egyptian Health Council, established in 2022, is a public service body mandated to develop and implement evidence-based national guidelines and reports to the Egyptian President.^7^ The council includes the Higher Committee for Guidelines, which identified priority topics for the national guidelines and supervised the development and adaptation of the guidelines; its members represent national stakeholders. The Ministry of Health and Population set the agenda as a policy priority and ensured engagement with national stakeholders.

To inform programme development, WHO conducted a situation analysis in November 2022. The analysis comprised a desk review of existing regulations, processes and published guidelines, together with a series of structured meetings with stakeholders and other key informants (Box 1). Each meeting followed a prepared template to assess existing processes, technical capacities and challenges as well as priorities for action for the development, adaptation, adoption and implementation of the guidelines.

Box 1 **Stakeholders engaged in the situation analysis for the roadmap in Egypt, November 2022**

- Ministry of Health and Population: 3 participants
- Central Administration for Critical and Urgent Care: 1 participant
- Chamber of Health Service Providers in the Private Sector: 2 participants
- Egyptian Drug Authority: 1 participant
- Egypt Healthcare Authority: 2 participants
- General Authority for Healthcare Accreditation and Regulation: 1 participant
- General Organisation for Teaching Hospitals and Institutes: 5 participants
- Medical Services Sector at the Ministry of Interior: 6 participants
- Ministry of Health and Population: 3 participants
- Ministry of Defence: 1 participant
- Presidential Initiative to End Waiting Lists: 2 participants
- Professional societies (Egyptian Society of Cardiology, Egyptian Otorhinolaryngology Society, Egypt Society of Obstetrics and Gynaecology, Egyptian Society of Nephrology and Transplantation, Society for Diagnostic Radiology, Egyptian Society of Neurological Surgeons, Egyptian Association of Urology, Egyptian Society of Chest Diseases and Tuberculosis, Egyptian Society of Laboratory Medicine and Egyptian Orthopaedic Association): 11 participants
- Supreme Council of University Hospitals: 1 participant

Total: 39 participantsNote: numbers from a situation analysis conducted by the World Health Organization, compiled by the authors.

The situation analysis identified several challenges, including: (i) different specialty societies, university departments and hospitals had produced or adapted guidelines using inconsistent methods; (ii) most institutions had limited or no technical capacity to use the GRADE (Grading of Recommendations, Assessment, Development and Evaluation) standards to develop or adapt guidelines; (iii) nursing and allied health professionals were rarely involved in clinical care guideline panels and produced their guidelines without consulting clinicians or other disciplines; (iv) there was no formal process for engaging the private sector; and (v) there was no central system to monitor guideline implementation, nor was there a national compendium of existing guidelines. The analysis also identified strengths and opportunities: (i) high-level support for evidence-based practice gave the programme visibility and helped secure budget allocation; (ii) the council provided a structure for streamlining the development and adaptation of the national guidelines; and (iii) parallel processes for hospital accreditation and health technology assessment programmes provided an opportunity to link the use of national guidelines to the requirements of these programmes. 

Following the situation analysis, WHO used the findings to support the establishment of the National Programme for Guideline Development and Adaptation. The support involved designing and conducting technical capacity-building workshops on guideline development and adaptation for stakeholders and creating a national roadmap for the guideline programme.

### Technical capacity-building

WHO organized two workshops in November 2022 and February 2023 for senior decision-makers from stakeholder organizations (Box 1) and key technical teams. Further capacity-building events in October and November 2023 focused on those involved in developing or adapting the guidelines and covered GRADE methods and tools. The events covered the methods for developing and adapting guidelines, such as priority-setting, scoping, assessing existing guidelines, finding and appraising evidence, use of cost data, developing evidence-to-decision tables, formulating recommendations and decision-making processes of the guideline development group (Table 1).^2^^,^^8^^–^^10^

**Table 1:** Capacity-strengthening activities for stakeholders engaged in guideline development in Egypt

Workshop title	Date	Objective	No. of participants
**Workshops before the launch of the programme**			
High-level workshop on development, revision and implementation of the National Guideline Development and Adaptation Programme	22–23 Nov 2022	To bring together a group of national policy-makers and experts to review and discuss the country experience in the development, adoption and adaptation of guidelines, including challenges and implementation of guidelines in practice; agree on the main action points for establishing the national programme for development and adaptation of guidelines in Egypt	55
Technical workshop on the National Programme for Guideline Development and Adaptation	30 Jan–2 Feb 2023	To enhance the technical capacity for development and adaptation of guidelines and to conduct training of trainers for development and adaptation of the guidelines	60
Technical capacity-building workshop on GRADE methodology	9–11 Oct 2023	To enhance participants’ knowledge and practical skills to develop and adapt clinical guidelines, including prioritizing topics and questions, conducting and critically appraising evidence searches, assessing the quality and certainty of evidence using the GRADE methods and preparing structured evidence tables using GRADEpro software	50
Virtual technical capacity-building workshop on GRADE methodology	6–8 Nov 2023	To strengthen participants’ understanding of the GRADE-ADOLOPMENT methods and guideline statement types and build their capacity to assess health effects and contextual factors, gather relevant information and effectively use evidence-to-decision and adolopment tables within GRADEpro software	60
**WHO-supported training after the launch of the programme**			
Virtual guideline methodologist training	22–24 Apr 2025	To build the capacity of national methodologists to understand and apply the full process of guideline development and adaptation using the GRADE approach and effectively contribute to national guideline development and adaptation in alignment with international standards	21
2nd national training workshop for methodologists on guideline adaptation using GRADE methodology	11–12 Aug 2025	To build a sustainable cadre of national methodologists to support guideline development in Egypt	20

GRADE: grading of recommendations, assessment, development and evaluation.

Note: numbers are from registration information at the capacity building events, compiled by the authors.

In addition to building capacity, the workshops were designed to increase awareness among key policy actors on the importance of the role and use of evidence-based guidelines in service delivery, and the technical and procedural requirements for the adaptation and development of evidence-based guidelines.

### National roadmap for guidelines

WHO, in consultation with the health council, developed the roadmap for the National Programme for Guideline Development and Adaptation in October 2023. The roadmap outlined key steps in adaptation of evidence-based guidelines to the national context, the roles and responsibilities of key stakeholders (Table 2) and standard operating procedures for guideline development groups that should be established for each guideline.^7^ The roadmap also specified criteria for setting guideline priorities, principles for documentation, a template for drafting national guidelines, minimum criteria for implementation plans and strategies for monitoring and evaluation (Table 2). Additionally, WHO developed a plan for longer-term technical capacity-building to ensure the sustainability of the programme in Egypt, using national expertise.

**Table 2:** Key specifications of the roadmap for the National Programme for Guideline Development and Adaptation in Egypt, October 2023^7^

Main step	Required functions and responsibilities	Responsible entity
Identifying key stakeholders for the programme	Defining roles and responsibilities	Egyptian Health Council
Priority-setting for guidelines	Identifying priority topics using the minimum criteria: • Health and cost burden of the disease; • Practice variation or lack of established policies; • Potential impact to improve health outcomes	Higher Committee for Guidelines
Scoping the guidelines: questions to be addressed	Guidelines should include: • Guideline target group, such as patients and professionals; • Care setting, such as public health and primary care; • Aspects of care affected, such as promotion, prevention, diagnosis and treatment	Higher Committee for Guidelines; Guideline development groups
Establishing the guideline development groups and technical support teams	Establishing roles and responsibilities; Ensuring multidisciplinary participation, such as both clinicians and nurses; Managing conflicts of interest	Higher Committee for Guidelines; Egyptian Health Council
Writing guidelines	Ensuring standardization, clarity and ease of use	Guideline development groups; Egyptian Health Council (for approval and publishing)
Ensuring implementation plan, monitoring and evaluation plan for each guideline are in place and followed	Identifying potential barriers and facilitators; Noting the availability of resources; Developing implementation strategies; Establishing performance indicators, such as for guideline dissemination, end-user knowledge, delivery of care and health outcomes	Egyptian Health Council; other key stakeholders, including the Ministry of Health and Population, specialty societies, teaching hospitals and the private sector

Note: roadmap for the National Programme for Guideline Development and Adaptation, compiled by the authors.

## Relevant changes

In total, 225 individuals from more than 20 national stakeholders attended the capacity-building workshops. After the workshops, these individuals started applying the taught methods and approaches for guideline development and adaptation. The workshops built a common understanding of the approaches among stakeholders, including clinicians, nurses, allied health professionals and the private sector. The workshops also generated wider political support for the programme and empowered national experts and methodologists to engage with it.^8^

The council ratified the roadmap and in July 2024 the ministry, WHO and the council formally launched the programme. Implementation of the programme led to clearer delineation of responsibilities (e.g. for those developing and ratifying new guidelines), eliminated role overlaps (e.g. between the ministry and the council) and harmonized processes across different professional societies, teaching hospitals, nurses and other health workers.

The programme resulted in the publication of 49 clinical practice and public health guidelines between 2024 and February 2026, available in the online repository.^7^ Further plans are in place to review progress in 2026. The roadmap developed for Egypt’s national guideline programme also served as the basis for a generic guide for countries in similar situations.^11^

## Lessons learnt

The establishment of Egypt’s national guideline programme followed a multidisciplinary, collaborative approach, involving stakeholders from various ministries, institutions, regulatory agencies, professional societies and WHO. Political commitment at the highest levels was a key factor in the success of the agenda, as observed in Kenya and Saudi Arabia.^12^^,^^13^ While policy-makers often support guidelines to enhance care and efficiency, they may not welcome the limitations that a national guideline programme imposes on their decision-making power. They may also be hesitant to engage professional societies that wish to develop, adapt or adopt clinical guidelines without oversight from a national programme. In Egypt, national policy-makers established the vision and demand for the programme, driving the agenda forward and ensuring stakeholder buy-in.

Another important lesson was the need to establish institutional capacity and define roles. National guideline programmes cannot rely on external expertise, so the programme prioritized strengthening national technical capacity. The workshops addressed the shortage of local methodologists identified in the situation analysis and reported in similar settings.^14^ Legislation integrating the programme into the Egyptian Health Council helped align those stakeholders who did not report to the ministry. The integration bridged divides among teaching, military, police and insurance sector hospitals and facilitated engagement with private sector physicians. The council addressed key challenges, including funding constraints and inadequate standards, technical staff and infrastructure, by establishing a dedicated executive team and delivering extensive training (Box 2).

Box 2 **Summary of main lessons learnt**

- Multidisciplinary engagement and strong political commitment are essential for successful national guideline programmes.
- Training dedicated teams and building technical capacity for guideline development and adaptation is vital.
- Effective implementation requires tailoring guideline adaptation processes to country-specific needs, especially in low-resource settings.

WHO and the national stakeholders also identified the importance of tailoring the process to the context. Development of the national roadmap was a major contributor to enabling the establishment of the programme.^5^^,^^15^ Although international frameworks and tools offer guidance on national agendas, they often overlook the specific needs and challenges faced by low- and middle-income countries. This initiative produced a roadmap, in line with the experiences in other countries with similar contexts and broader critiques of global frameworks, while outlining and contextualizing the standard operating procedures to the Egyptian context.^16^ The roadmap avoided technical details for each step, as existing guidance already covers these aspects.^2^

The collaboration among WHO’s country, regional and headquarters offices complemented each other and supported the programme. The WHO country office served as the focal point, coordinating the programme between the ministry, the council and WHO. The regional office provided hands-on strategic and technical support and mobilized international resources. WHO headquarters provided further technical support when needed. 

Existing programme resources covered the operational costs, totalling about 40 000 United States dollars, including organization of training activities and engagement of external trainers.

Moving forward, monitoring and evaluating the development and adaptation processes, as well as the impact of the guidelines, are essential. Ensuring sustained funding, capacity-building, stakeholder engagement and strong governance structures will be vital to the programme's long-term success. Sharing these experiences and lessons can benefit other countries or organizations in establishing similar national guideline programmes and improving health-care systems worldwide.^8^^,^^11^
